# Influence of Sinogram Affirmed Iterative Reconstruction of CT Data on Image Noise Characteristics and Low-Contrast Detectability: An Objective Approach

**DOI:** 10.1371/journal.pone.0056875

**Published:** 2013-02-13

**Authors:** Christian von Falck, Vesela Bratanova, Thomas Rodt, Bernhard Meyer, Stephan Waldeck, Frank Wacker, Hoen-oh Shin

**Affiliations:** 1 Department of Diagnostic and Interventional Radiology, Hannover Medical School, Hannover, Germany; 2 Department of Radiology, Bundeswehrzentralkrankenhaus, Koblenz, Germany; University of Adelaide, Australia

## Abstract

**Objectives:**

To utilize a novel objective approach combining a software phantom and an image quality metric to systematically evaluate the influence of sinogram affirmed iterative reconstruction (SAFIRE) of multidetector computed tomography (MDCT) data on image noise characteristics and low-contrast detectability (LCD).

**Materials and Methods:**

A low-contrast and a high-contrast phantom were examined on a 128-slice scanner at different dose levels. The datasets were reconstructed using filtered back projection (FBP) and SAFIRE and virtual low-contrast lesions (-20HU) were inserted. LCD was evaluated using the multiscale structural similarity index (MS-SIM*). Image noise texture and spatial resolution were objectively evaluated.

**Results:**

The use of SAFIRE led to an improvement of LCD for all dose levels and lesions sizes. The relative improvement of LCD was inversely related to the dose level, declining from 208%(±37%), 259%(±30%) and 309%(±35%) at 25mAs to 106%(±6%), 119%(±9%) and 123%(±8%) at 200mAs for SAFIRE filter strengths of 1, 3 and 5 (p<0.05). SAFIRE reached at least the LCD of FBP at a relative dose of 50%. There was no statistically significant difference in spatial resolution. The use of SAFIRE led to coarser image noise granularity.

**Conclusion:**

A novel objective approach combining a software phantom and the MS-SSIM* image quality metric was used to analyze the detectability of virtual low-contrast lesions against the background of image noise as created using SAFIRE in comparison to filtered back-projection. We found, that image noise characteristics using SAFIRE at 50% dose were comparable to the use of FBP at 100% dose with respect to lesion detectability. The unfamiliar imaging appearance of iteratively reconstructed datasets may in part be explained by a different, coarser noise characteristic as demonstrated by a granulometric analysis.

## Introduction

The use of computed tomography (CT) has increased dramatically during the last two decades, accelerated by the rapid technical improvement with milestones such as the introduction of spiral scanning and multidetector CT (MDCT). Although CT examinations account for approximately 17% of all radiological examinations using ionizing radiation in the US, the collective efficient dose of all CT examinations amounts to almost half of the total patient exposure [1]. Comparable data is available for the situation in the Germany [2]. To date, the potential risk of radiation exposure from CT cannot be reasonably estimated on a patient or even examination base. However, recent publications suggest a significant increase in lifetime cancer risk from CT examinations and have led to increasing dose awareness, not only in the radiological but also in the general medical community [3].

One approach towards dose reduction in CT imaging is the iterative reconstruction of image data instead of using the traditional filtered back projection algorithms. Iterative reconstruction algorithms are well established in the field of nuclear medicine as they allow the generation of diagnostic images even from statistically poor raw data. However, their routine use for CT reconstruction had been limited by the availability of sufficient computing power that enables a reconstruction time suitable for clinical use [4]. Only recently, several manufacturers have introduced proprietary iterative reconstruction algorithms in their commercially available scanners and suggest a significant improvement in the signal-to-noise ratio, allowing for dose-wise scanning protocols [5] while retaining the detectability of low-contrast objects.

The aim of this study is to systematically investigate the influence of the sinogram affirmed iterative reconstruction algorithm (SAFIRE) on image noise characteristics and low-contrast performance using an objective approach that combines software-generated ‘virtual’ lesions with an image quality metric.

## Materials and Methods

For the objective evaluation of low-contrast detectability we utilized a novel approach that combines a software phantom capable of generating virtual lesions in CT datasets with a full-reference image quality metric [6]. In brief, a homogenous physical phantom is examined on an MDCT scanner and image data is reconstructed. A second dataset is created by adding a single virtual low-contrast lesion within this original dataset. As these two dataset are completely similar except for the simulated lesion, a full-reference image quality metric can now be used to predict the visible difference between the two datasets. This difference can be regarded as a surrogate marker for lesion detectability [6].

### Phantom design

**Figure 1:**
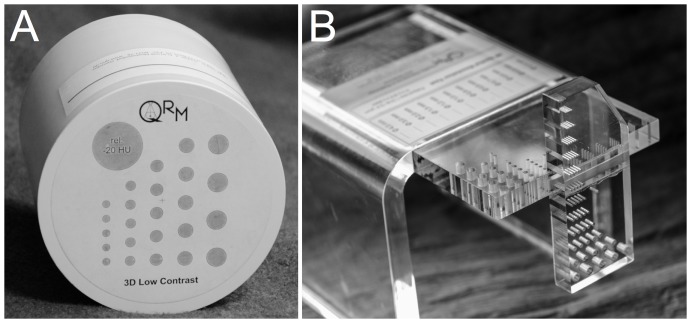
Photograph of the 3D low-contrast phantom (a) and the 3D spatial resolution phantom (b).

A low-contrast phantom designed for MDCT (QRM GmbH, Moehrendorf, Germany) was used to simulate the lesion-free background of parenchymal organs (Fig. 1a). The average CT value of the homogenous background medium of the phantom was 35HU. Another part of the phantom contained spheres of different diameters ranging from 3 mm to 8 mm simulating hypodense lesion with density of 15HU, and a 2.0 cm calibration cylinder. This part of the phantom was only used to measure the consistency of the HU values throughout the study.

A high-contrast 3D resolution phantom for MDCT (QRM, Moehrendorf, Germany) was used to evaluate the influence of the difference reconstruction algorithms on spatial resolution (Fig. 1b). The phantom consisted of a PMMA (polymethyl methacrylate) slab containing a test pattern of cylindrical drill holes with varying diameter and spacing ranging from 4 mm to 0.4 mm.

### Scan and reconstruction parameters

The phantoms were placed in a row on the CT table at the isocenter of a 128-slice MDCT scanner (Somatom Definition Flash, Siemens, Forchheim, Germany). The scan parameters are given in detail in Table 1. In brief, the phantoms were scanned at five different dose-levels of 25mAs, 50mAs, 100mAs, 150mAs and 200mAs with a constant tube current (no dose modulation) at a tube voltage of 120kVp. The images were reconstructed using the filtered back projection algorithm with a standard abdomen kernel (B30f) as well as using the corresponding iterative back projection algorithm (SAFIRE I30f) with three different filter strengths (I1, I3, I5). A total of 20 DICOM datasets were created.

**Table 1:** Image acquisition and reconstruction parameters.

Acquisition parameters	
CT scanner	Somatom Definition Flash (Siemens Healthcare, Forchheim, Germany)
Tube voltage	120kVp
Tube current – time product	25mAs, 50mAs, 100mAs, 150mAs, 200mAs (no dose modulation)
Rotation time	0.5s
Slice collimation	0.6 mm
Pitch	0.6
Reconstruction parameters	
Reconstructed slice thickness	0.75 mm
Reconstruction interval	0.50 mm
Matrix	512x512px
dFOV	256 mm
Reconstruction algorithm FBP	B30f
Reconstruction algorithm SAFIRE	I30f 1, I30f 3, I30f 5

The SAFIRE algorithm is based on a hybrid iterative approach, combining a raw data iterative approach with an image-based iteration loop. In the raw data (sinogram) domain, primarily reconstructed ‘draft’ FBP images undergo forward projection while taking into account the relevant scanner geometry. The synthetic raw data is then compared with the measured raw data. The differences identified in this step are used for raw data correction in the subsequent iteration. This step is primarily used to eliminate artifacts from the reconstruction such as spiral and cone-beam artifacts. Within each iteration, a model-based noise reduction is applied. The second iteration loop operates in the image domain and uses model-based noise reduction to improve image quality. While noise reduction has been shown to be mathematically equal in both domains, the operation in the image domain is much less time consuming but does not reduce reconstruction artifacts. Therefore, the SAFIRE algorithm combines both approaches to enable high-quality iterative reconstruction in a reasonable processing time.

### Lesion simulation using a ‘software phantom’

**Figure 2:**
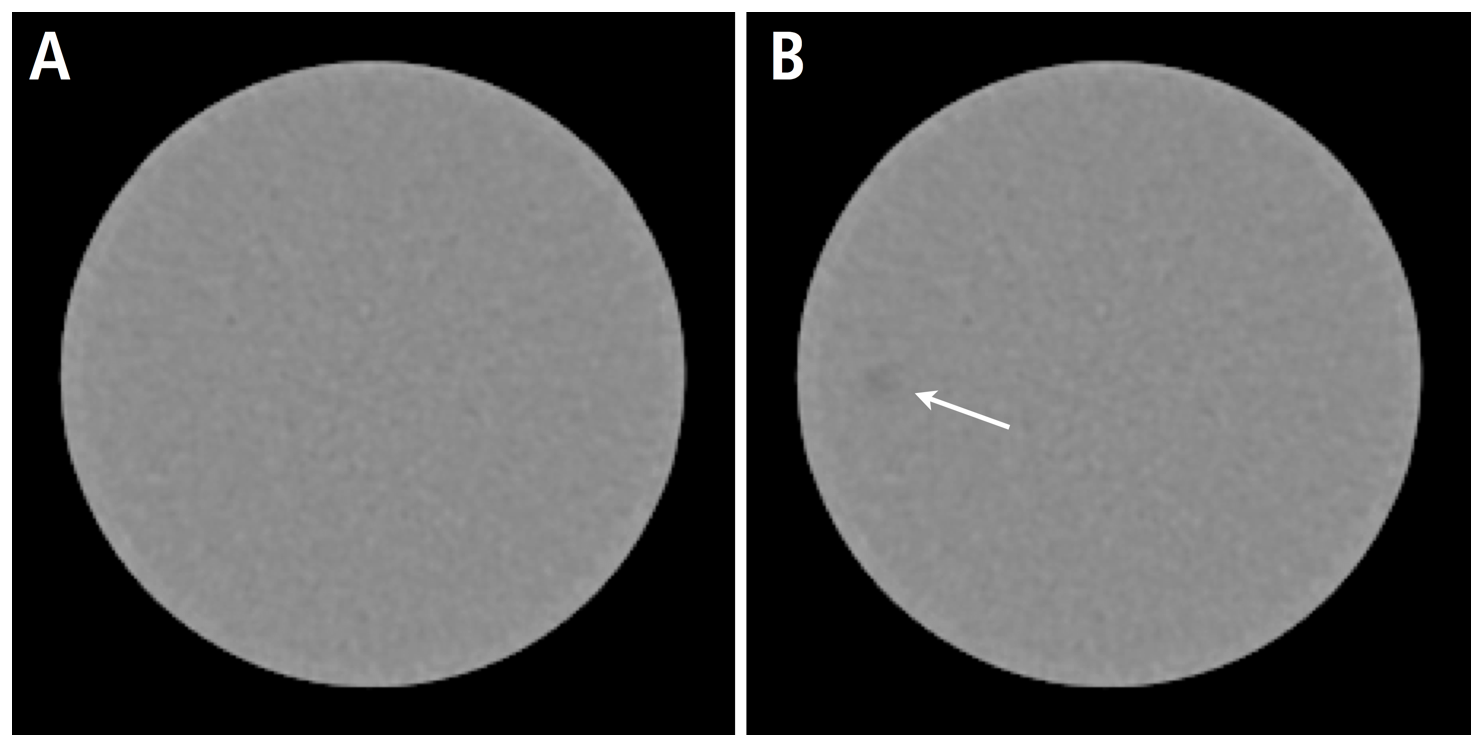
Insertion of virtual lesions. Exemplary illustration of an axial slice through the low contrast phantom before (a) and after (b) the insertion of a 5 mm virtual lesion in a background image acquired at 100mAs and reconstructed with the I30f algorithm with a filter strength of 3.

A ‘software phantom’ was developed using MeVisLab 2.2, a modular framework for medical image processing and visualization as previously described [6], [7]. Briefly summarized, a library of three-dimensional low-contrast lesions is created by segmenting poorly contrasted lesions from actual CT datasets in a preparatory step. These segmented lesions can then be ‘added’ to any target CT DICOM dataset using an alpha blending technique, positioned with a single mouse-click at the discretion of the user. The size and the density of the virtual lesions can be adjusted to match the desired object contrast and dimension by modifying a weighting and a scaling factor. If the internal structure of the segmented lesion is switched off in the software phantom as done in this study and the weighting factor is small, the virtual lesion inherits the noise magnitude and characteristics of the target datasets to a large extent. The resulting dataset containing the virtual lesion can now be exported in the DICOM format and post-processed or analyzed as favoured. Figure 2 shows an example of a virtual lesion.

We used this ‘software phantom’ approach to insert virtual hypodense low-contrast lesions with a contrast of -20HU in the CT datasets acquired and reconstructed as shown above. The size of the lesions was systematically increased from 4 mm to 5 mm, 6 mm, 7 mm, 8 mm and 10 mm (1–6) and five different lesions of the same size were inserted at different standardized positions. Each time, only a single virtual lesion was added to the background-only part of the low-contrast phantom of each dataset at a predefined position. The slice at the centre of the virtual lesion was exported as a single DICOM image. Finally, lesion-free images were generated for each of the 20 datasets at the same z-position. Thereby, a total of 620 DICOM images (5 lesions x 6 lesion sizes x 5 dose levels x 4 reconstructions + 20 lesion free datasets) were created for the following analysis.

### Image quality metric (MS-SSIM*)

The multiscale structural similarity index (MS-SSIM) [8] in the version as optimized by Rouse and Hemami (MS-SSIM*) [9] for the recognition threshold problem was used to calculate the visibly perceivable difference between the images containing the virtual lesions and their corresponding lesion-free counterparts, expressed as the structural similarity index. A high MS-SSIM* index indicates a high degree of perceived similarity between the images. As both images only differ by the lesion that is included in one of them, this similarity index can be regarded as a surrogate parameter for lesion detectability [6].

For the purpose of this study we used the Java implementation of the algorithm that has been developed by Prieto et al. and is available as a plug-in for the open-source image processing and analysis software ImageJ 1.44o [10]. In practice, each DICOM image containing a virtual lesion was imported into ImageJ together with its lesion-free counterpart, i.e. the background-only image acquired at the same dose and reconstructed with the same algorithm. Contrast and brightness of both images were adjusted according to a standard abdomen window (center = 40HU, width = 350HU) and the above mentioned plug-in was used to calculate the MS-SSIM* index. For every dataset the results of same-sized lesions were averaged. The differences between the datasets were tested for statistical significance using the unpaired student’s t-test.

### Image noise analysis

The influence of the different dose setting and reconstruction algorithms on the magnitude of the image noise was analyzed by calculating the mean and the standard deviation of the HU-values as measured in a square ROI (area = 900 mm^2^) placed on the lesion-free background-only image. The measurement was repeated four times per dataset (at different predefined coordinates) and the averages and standard deviations were determined.

The size distribution as a measure of the perceived granularity of the image noise was determined using granulometry, implemented as a plugin for the open-source image processing and analysis software ImageJ 1.46 [11]. In a first step, the intensity contrast of a noise image was maximized using binary thresholding based on the isodata algorithm [12]. Second, the size distribution of the image noise was estimated as a function of the opening size. This approach is comparable to sifting the grainy structures of the background noise using screens of increasing mesh size. This analysis was performed for every background-only image.

### Assessment of spatial resolution

For the assessment of possible differences in spatial resolution the image analysis software ImageJ 1.46 was used to calculate line profiles along the 4 mm cylindrical drill holes on a slice centered on the resolution phantom in all datasets. The maximum slope of the profile as a measure of spatial resolution was calculated for all of the five lesions in each dataset and the results were averaged. The unpaired student’s t-test was used to evaluate possible statistically significant differences between the different datasets.

## Results

### Assessment of low-contrast detectability using the image quality metric

In general, the use of the iterative reconstruction algorithm led to an improvement in lesion detectability for all dose levels and all lesions sizes. The low-contrast performance was lowest for the standard filtered back projection approach at all dose levels and increases with increasing filter strength of the SAFIRE algorithm.

**Figure 3:**
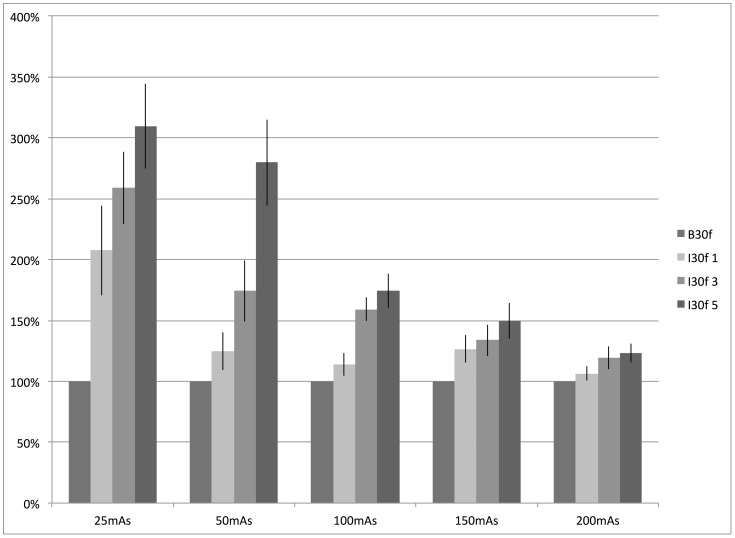
Improvement of low-contrast detectability depending on dose level. Graphical illustration of the relative improvement of low contrast detectability using the iterative reconstruction approach when compared with the FBP reconstruction depending on dose level. It can be appreciated that beneficial effect of the iterative approach is most prominent in datasets acquired at low dose levels and reconstructed with high filter strength.

However, we observed a dependency of the degree of the relative improvement of low-contrast performance on the dose level. Regarding the relative improvement of lesion visibility relating to the filtered back projection algorithm (B30f), the values declined from 208% (±37%), 259% (±30%) and 309% (±35%) at 25mAs down 106% (±6%), 119% (±9%) and 123% (±8%) at 200mAs for SAFIRE filter strengths of 1, 3 and 5, respectively. These dependencies are outlined in detail in Table 2 and illustrated in Figure 3. The differences with respect to the corresponding B30f dataset were considered statistically significant (p<0.05).

**Table 2:** Relative improvement of lesion visibility using iterative reconstruction relating to the filtered back projection algorithm.

	Dose level				
**Filter Strength**	**25mAs**	**50mAs**	**100mAs**	**150mAs**	**200mAs**
**I30f 1**	208% ±37%	125% ±15%	109% ±9%	127% ±11%	106% ±6%
**I30f 3**	259% ±30%	174% ±25%	159% ±10%	134% ±13%	119% ±9%
**I30f 5**	309% ±35%	280% ±35%	175% ±14%	150% ±15%	123% ±8%

**Figure 4:**
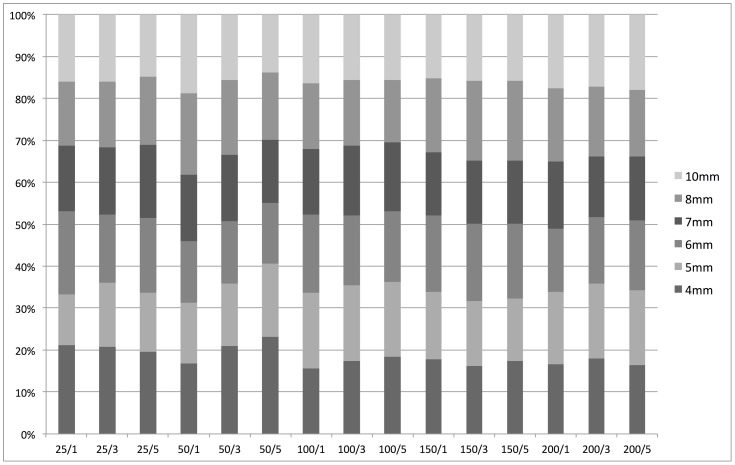
Improvement of low-contrast detectability depending on lesion size. This normalized stacked bar chart visualizes the relative improvement of low contrast detectability using the iterative reconstruction approach as compared to the FBP reconstruction depending on lesion size. The different dose levels as well as the filter strengths of the SAFIRE algorithm are given on the x-axis. The similar distribution of the stacked bars confirms that the relative improvement of low contrast detectability using the SAFIRE algorithm is vastly independent of the lesion size.

Interestingly, the beneficial effect of the iterative reconstruction algorithm showed only a minor dependency on lesion size as outlined in detail in Table 3 and illustrated in Fig. 4. The relative improvement of lesion detectability averaged over all dose levels as compared to the standard B30f reconstruction ranges from 120% (±65%), 154% (±81%), 183% (±85%) at 4 mm, to 120% (±38%), 142% (±51%) and 162% (±61%) at 10 mm for SAFIRE filter strengths of 1, 3 and 5, respectively. The differences cannot be considered statistically significant (p>0.05). The high standard deviations can be explained by the influence of the dose level as outlined above. However, it is clearly observable from the normalized stacked bar chart (Fig. 4) that the relative improvement of lesion detectability is widely independent of lesion size.

**Table 3:** Relative improvement of lesion detectability using iterative reconstruction averaged over all dose levels as compared to the standard B30f reconstruction.

	Lesion diameter					
Filter Strength	4 mm	5 mm	6 mm	7 mm	8 mm	10 mm
**I30f 1**	120% ±65%	118% ±18%	123% ±60%	112% ±38%	122% ±34%	120% ±38%
**I30f 3**	154% ±81%	145% ±46%	144% ±52%	136% ±56%	149% ±46%	142% ±51%
**I30f 5**	183% ±85%	170% ±74%	170% ±81%	158% ±87%	166% ±80%	162% ±61%

With regard to dose reduction, the datasets reconstructed using the iterative approach reached at least the low-contrast performance of the datasets reconstructed with the FBP algorithm at a relative dose reduction of 50%. The necessary filter levels varied with respect to the absolute dose: I5 at 25mAs and 50mAs and I3 at 100mAs.

### Image noise analysis

In general, the standard deviation of the HU-values of the background decreased with increasing dose level. It was lower for datasets reconstructed with the iterative approach as compared to the standard filtered back projection and was inversely relate to the filter strength. The noise levels ranged from 10.41HU (±0.35HU), 9.27HU (±0.32HU), 6.94 (±0.25HU) and 4.70HU (±0.18HU) at 25mAs down to 3.83HU (±0.07HU), 3.42HU (±0.06HU), 2.60HU (±0.04HU) and 1.84HU (0.03HU) at 200mAs for image reconstruction with the B30f, I30f 1, I30f 3 and I30f 5 algorithm, respectively. The results are outlined in detail in Table 4.

**Table 4:** Noise level of phantom background dependent on reconstruction algorithm and dose level.

	Dose level				
Kernel	25mAs	50mAs	100mAs	150mAs	200mAs
	[HU]	[HU]	[HU]	[HU]	[HU]
B30f	10.41 ±0.35	7.32 ±0.24	5.29 ±0.08	4.38 ±0.22	3.83 ±0.07
I30f 1	9.27 ±0.32	6.51 ±0.21	4.71 ±0.08	3.91 ±0.20	3.42 ±0.06
I30f 3	6.94 ±0.25	4.89 ±0.17	3.56 ±0.06	2.97 ±0.13	2.60 ±0.04
I30f 5	4.70 ±0.18	3.35 ±0.13	2.44 ±0.07	2.07 ±0.08	1.84 ±0.03

The mean CT value of the four background ROIs averaged over all datasets was 33.41HU (±0.22HU). There was no statistically significant difference of the mean CT value of the background ROIs in the different datasets (p>0.05). The object contrast was therefore regarded as constant over all datasets and the inverse of the standard deviation of the background (as calculated above) can be interpreted as the signal-to-noise ratio (SNR). The relative improvement of this SNR as compared to the respective B30f reconstructions averaged over all dose levels reached 112.24% (±0.19%), 148.67% (±1.19%) and 215.36% (±5.41%) for reconstructions with the I30f 1, I30f 3 and I30f 5 algorithm, respectively. Interestingly, there were no statistically significant differences of this relative improvement of the SNR for the different dose levels (p>0.05).

**Figure 5:**
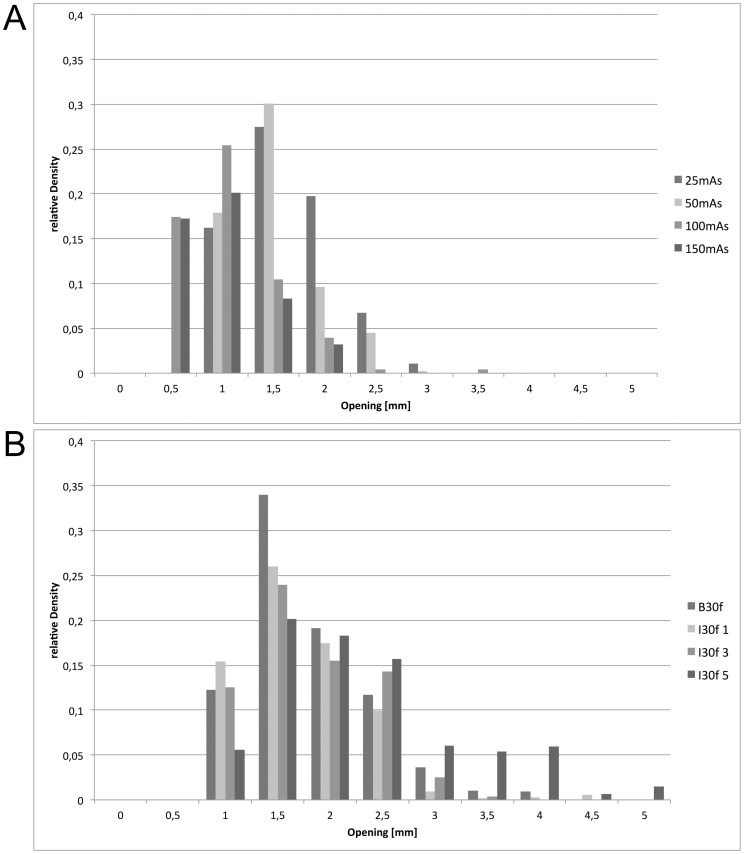
Analysis of image noise characteristics. Analysis of the image noise characteristics as determined using a granulometric analysis for the FBP reconstruction at different dose levels (a) and the iterative reconstruction using different filter strengths at 200 mAs (b). The opening radius of the granulometric algorithm as given on the x-axis can be regarded as a descriptor of the granularity of image noise. It can be observed that there is a minor shift towards finer image noise granularity with increasing dose setting (a). In contrast, the use of the iterative reconstruction algorithm leads to a shift towards a coarser granularity with an opening radius higher than 3 mm, especially for the highest filter strength.

**Figure 6:**
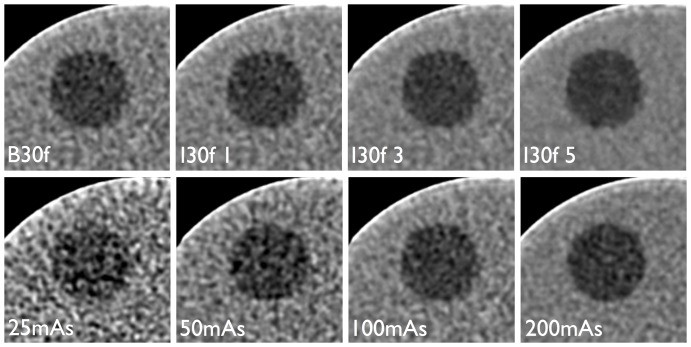
Illustration of the different image noise characteristics. Illustration of the different noise characteristics using the iterative algorithm ‘I30f’ with increasing filter strength opposed to the standard FBP reconstruction ‘B30f’ (top row) in comparison to the effects of an increasing dose level on standard FBP reconstruction (B30f, bottom row). The relatively high contrast between the lesion and the background results from the very narrow windows that was chosen was illustrative purposes. Although overall image noise decreases from left to right in both rows, there is a clear shift towards a coarser noise granularity using the iterative reconstruction (top row) whereas almost similar noise characteristics are observed for increasing dose levels of the FBP reconstruction (bottom row).

We analyzed the influence of the applied dose and the reconstruction algorithm on the granularity of image noise using the granulometric approach as described above. The increase of the tube current time product from 25mAs to 50mAs, 100mAs and 150mAs led to a discrete shift of the dominant noise granularity towards smaller clusters. The most frequently observed opening sizes were 1.5 mm and 2 mm at 25mAs, 1.0 mm and 1.5 mm at 50mAs and 0.5 and 1.0 at 100mAs and 150mAs, respectively. Interestingly, the use of the iterative reconstruction algorithm had the opposite effect with a shift of the image noise granularity towards larger clusters with an opening of 3 mm to 5 mm, especially when the maximum filter strength (I5) was applied. These findings are summarized in Figures 5a/b and illustrated in Figure 6 and may help understand the “blocky” appearance of iteratively reconstructed CT images as repeatedly described in the literature [5], [13], [14].

### Spatial resolution

The mean slope of the line profile over the five drill holes averaged over all datasets was -1002.71 HU/mm (±5.32 HU/mm). For each dose level, there was no significant difference between the results for the standard filtered back projection and the iterative algorithm with different filter levels of 1, 3 and 5, respectively (p>0.05). For each of the different reconstructions algorithms, there was no statistically significant difference between the results for the different dose levels, as well (p>0.05). The images of the spatial resolution phantom for the different dose levels and reconstruction algorithms are summarized in Fig. S1 for illustrative purposes.

## Discussion

Modern MDCT is well appreciated as a highly valuable tool in clinical routine imaging with decisive importance on patient diagnosis and management in diverse medical fields such as oncology, traumatology and cardiovascular disease. However, CT is associated with a significant radiation burden to the patient and is the major contributor to the cumulative radiation exposure resulting from medical imaging procedures [1], [2]. Although the probability of cancer induction resulting from ionizing radiation cannot be readily calculated on an examination-base, there has grown statistical evidence of potentially fatal side effects of CT imaging [3]. The increasing dose awareness in the radiological and the general medical community has driven radiologists as well as equipment manufacturers to optimize CT imaging procedures with respect to dose reduction. A major advancement in device-related dose reduction after the widespread adoption of dose modulation techniques is the recent introduction of iterative reconstruction algorithms by all manufacturers [5]. The basic principle of iterative reconstruction is well established since the 1960s and such algorithms have been routinely use for the reconstruction of images from nuclear medicine data, which is general more prone to severe image noise in case of poor counting statistics [4], [5]. However, such algorithms have been impractical for the use on CT data for years. Only recently, the availability of sufficient computing power using affordable standard hardware has enabled the implementation of iterative reconstruction algorithms for routine use [5]. Unfortunately, the algorithms implemented on commercially available CT scanners are highly vendor- and device-specific as they take into account detailed scanner information such as the geometry and the algorithms therefore rather appear as ‘black-boxes’ [5]. There is growing evidence in the radiological literature that these algorithms may allow a substantial decrease in the necessary radiation exposure to the patient while retaining diagnostic image quality [15]. Basically, changes in image noise are critical in MDCT as image noise is directly related to the detectability of low-contrast lesions, a highly relevant scenario in clinical CT imaging, e.g. for the detection of metastases in parenchymal organs [16].

However, published data on the effects of the proprietary iterative algorithms on low-contrast detectability is mainly based on rather simple image quality measures such as the signal-to-noise ratio or on subjective image analysis based on reader studies, which are known to suffer from relevant shortcomings. Indeed, the SNR is a poor measure of lesion detectability and does not correlate well with the judgment of human readers [16]. Such reader studies are still regarded gold standard, as they are comparable to the clinical reading situation, especially when they are based on real patient data. However, they are prone to substantial methodical drawbacks. First and most obvious, they may be impractical to perform in large studies with hundreds to thousands of images to be read. In particular, during the development and evaluation of novel algorithms for reconstruction or post-processing with many parameters to be adjusted, there is a need for a rapid and automated evaluation of image quality, before the pre-optimized datasets can be finally judged by human readers. Notably, objective analysis of image quality without human readers is daily routine in many fields such as still image and video compression, e.g. for the transmission of digital TV signals. Furthermore, results are dependent on the reader’s personal preferences, experience and alertness at the time of reading. Changes in the overall image appearance, e.g. a higher overall noise level or a different noise characteristic, may strongly influence the reader’s judgment, which is of particular interest in the evaluation of novel reconstruction algorithms with their unfamiliar image ‘impression’. In phantom studies, the known fixed arrangement of the lesions can bias the results, as well [4].

In this study, we therefore aimed at the objective evaluation of the detectability of low contrast lesions against the background of image noise as resulting from the iterative reconstruction approach in comparison to filtered back projection. The combination of a virtual software phantom and a full-reference image quality metric for the evaluation of low-contrast detectability has been recently described [6]. This approach yields several benefits: First of all, the evaluation process can be highly automated and allows the rapid analysis of large amounts of data. Notably, the use of a virtual phantom is vital to this approach. It enables the use of full-reference image quality metrics, as two almost similar datasets can be generated that only differ by the low-contrast lesion to be analyzed. This would no be possible while using a physical phantom, only. Second, this approach is highly flexible as it allows the virtual lesions to be tailored according to the specific need of the study with respect to lesion size, density, structure, shape and location in three dimensions. The lesions can be either derived from real CT-data or created completely artificial. The virtual phantom might in principle even be used for other imaging modalities such as MRI. Third, objective image quality assessment is insensitive to potential bias from an unfamiliar image appearances as observed with iterative reconstruction algorithms [4], [14]. Fourth, the objective approach used herein relies on well-established techniques and can therefore be regarded as robust. Moreover, it can easily be adopted for comparable studies and it can readily be modified, e.g. in order to participate in the latest developments in image quality metric algorithms.

A possible restriction of this approach in the evaluation of novel reconstruction algorithms is that the reconstruction algorithm does not directly affect the reconstruction of the lesion and therefore not all effects of the reconstruction algorithm are simulated (e.g. with respect to lesion border definition). However, as we use an alpha blending approach with a very low opacity setting for lesion insertion, the simulated lesions inherit the noise magnitude and characteristics of the target dataset to a large extent. Moreover, image noise magnitude and characteristics of the background can be regarded as the predominant determiners of the detectability of low contrast lesions and the actual structure of the lesion is of minor importance [17]. Hoe et al. even use a radially symmetric lesion model for the evaluation lesion detectability, that is more artificial than our approach [18]. Moreover, we regard the preservation of the original noise of the target dataset as superior to an imperfect mathematical modeling of image noise as otherwise reported [18], [19]. Our approach could be even extended to a complete evaluation of all features of the reconstruction algorithm if the virtual lesions were created in the scanners raw data domain before reconstruction. However, access to raw data and scanner details is generally limited and requires close collaboration with the scanner’s manufacturer.

The beneficial effect of the iterative approach was most prominent in the dataset acquired at the lowest dose setting of 25mAs with a range of 208% – 309% (depending on filter strength), i.e. the dataset containing the strongest image noise. In datasets containing a lower overall image noise, the beneficial effect was less prominent, but still statistically significant. Interestingly, there was no statistically significant effect of the lesion size on the relative improvement of lesion detectability when using the iterative reconstruction algorithm. Our results are in good concordance with the previously published data and show a substantial increase in lesion detectability with the use of the iterative reconstruction with a possible dose reduction in the range of 50% without compromising lesion detectability. Baker et al. reported a decrease in image noise and an increase in SNR for the SAFIRE reconstruction (at filter strength 3) acquired at 50% dose when compared with an FBP reconstruction acquired at 100% [4]. Kalra et al. found no loss of image quality in abdominal CT images using SAFIRE with a dose reduction of 50 – 75% [20]. Qi and colleagues showed that a dose reduction of 57% using an iterative approach (ASIR, General Electric Healthcare, Chalfont St. Giles, UK) is feasible in CT imaging of the chest without compromising image quality [21]. May et al. found no deterioration of image quality in abdominal CT at 50% dose using an iterative reconstruction approach operating solely in the image domain (IRIS, Siemens Healthcare, Forchheim, Germany), the predecessor of the algorithm evaluated in our manuscript [22]. Opposed to simple filter-based approached for the reduction of image noise, the iterative reconstruction is supposed to retain the spatial resolution. Our results are well in concordance with this assumption, showing no significant deterioration of the spatial resolution in our datasets [5], [23].

We further analyzed the effects of the iterative approach on image noise quantity and quality. With respect to image noise quantity, we observed a mean relative reduction of the SNR of the background in the range of 112%, 149% and 215% when using the iterative approach at filter strengths of 1, 3 and 5, as expected. Opposed to the results of the lesion-detectability as outlined above, these effects were independent of the underlying noise level (i.e. dose level at acquisition) of the raw data. Clearly, noise magnitude alone is not a sufficient descriptor of the effects of iterative reconstruction. However, regarding the image noise quality, there is only sparse literature on the different visual appearance of CT data reconstructed with iterative algorithms as compared to the traditional filtered back projection approach. The effects are commonly only figuratively described as ‘blocky’, but not further characterized in an objective manner [20], [24]. The results obtained from the granulometric analysis of the image noise as performed in this study may add to the understanding of this effect. We observed a tendency towards a more coarse appearance of the image noise containing larger clusters of pixels in datasets reconstructed with the iterative approach as compared to filtered back projection, especially with the strongest filter setting (I30f 5) as previously noticed in the literature. Interestingly, when using FBP only, an increase in dose was associated with the opposite effect, i.e. a shift to a finer appearance of the image noise. Despite an increase in low-contrast performance with the possibility of dose reduction the unfamiliar appearance of the images reconstructed using iterative approaches may negatively influence radiologist’s acceptance of the method [4].

A number of further limitations to this study need to be acknowledged. First, our study included only one type of lesions with a hypodense appearance of -20HU. However, this object contrast allows lesion detection even at small size and lowest dose settings and has been described in the literature for comparable purposes [25], [26]. We intentionally limited our virtual lesions to spherical objects as round or spherical lesions are widely accepted surrogate structures for the evaluation of low-contrast detectability in reader or phantom studies [17], [18]. As a matter of fact, phantom studies cannot fully simulate the complex clinical reading and decision making process but rather focus on one aspect of image quality. The low-contrast detectability as evaluated in this manuscript using idealized spherical lesions is the indispensable pre-condition for clinical lesion detection and consecutive interpretation. Notably, the virtual lesions as created with this software phantom in CT datasets have been shown to be indistinguishable from real lesions by human readers [27].

Furthermore, as we compared the influence of iterative versus standard reconstruction and did not analyze absolute detection thresholds, the object contrast is of minor importance. For the same reason, we chose an overall noise level, that was lower than in typical clinical CT scanning, but enabled a statistically valid analysis of even the smallest lesions at lower dose settings.

Second, we only analyzed one reconstruction kernel using FBP (B30f) and compared it to three filter strengths of one iterative algorithm (I30f). However, B30f is regarded as the standard abdomen kernel and the chosen iterative kernel has been designed by the manufacturer to be comparable to the standard B30f algorithm. Furthermore, we covered a broad spectrum of noise intensities by applying different dose levels from 25mAs to 200mAs and therefore assume our results to be valid for other kernel settings, as well.

Third, we did not compare our results with a reader study. However, the high number of lesions (*n = *600) analyzed in this phantom study rendered a reader study impracticable. In addition, as already outlined above, the ‘unusual’ appearance of iteratively reconstructed CT may be regarded as a possible confounder during the assessment of lesion detectability as radiologists are highly adapted to certain image characteristics. Furthermore, the approach of combining a virtual phantom and the MS-SSIM* metric has been described earlier for comparable purposes and correlated well with the judgment of human readers [6]. The MS-SSIM algorithm is based on the assumption that the human visual system has evolved in continuous response to the statistical regularities of our physical surrounding and measures visual quality by measuring differences to this statistical reference [6], [8], [9]. It therefore evaluates features such as luminance, contrast and structure of a test image in comparison to a given reference image. We have chosen the modified version of this specific algorithm, as it closely matches the near-threshold detection task of our study [8], [9]. Furthermore, it has proven to be accurate in medical and non-medical scenarios, as well [28]–[30]. We therefore regard our approach combining a software phantom with the MS-SSIM* metric as appropriate within the scope of this study. Moreover, our approach is highly flexible and offer the possibility to easily integrate novel image quality metrics that might be developed in the future if they outperform the MS-SSIM* metric [9].

Finally, we evaluated only one specific iterative reconstruction algorithm (SAFIRE). As these algorithms are highly vendor- and scanner-specific the results derived in this study cannot be transferred to iterative approaches as implemented by other manufacturers and no direct comparison of these algorithms has been published, so far. However, as described above the magnitude of dose reduction that is achievable with iterative reconstruction is roughly comparable for the different algorithms available on the market.

In conclusion, we used an objective approach combining a software phantom and the MS-SSIM* image quality metric to analyze the detectability of low-contrast lesions against the background of image noise as created using SAFIRE in comparison to filtered back-projection. We found, that lesion detectability using SAFIRE at 50% dose was comparable to the use of FBP at 100% dose. The unfamiliar imaging appearance of iteratively reconstructed datasets may in part be explained by a different, coarser noise characteristic as demonstrated by a granulometric analysis.

## Supporting Information

Figure S1 **Illustration of spatial resolution in different datasets.** Montage of axial slices through the spatial resolution phantom at the same z-position for all 20 datasets acquired at different dose levels and reconstructed using different algorithms and filter strengths. The visual assessment reveals no difference in spatial resolution between the different datasets as confirmed by the quantitative analysis. The reduction of image noise with increasing dose and increase filter strength of the SAFIRE algorithm can be noted in the homogeneous part of the phantom.
(TIF)
