# Supplementary figures and images for: Influence of Sinogram Affirmed Iterative Reconstruction of CT Data on Image Noise Characteristics and Low-Contrast Detectability: An Objective Approach
Source: PLoS One. 2013 Feb 13;8(2):e56875. doi: 10.1371/journal.pone.0056875 (PMC3572072; doi:10.1371/journal.pone.0056875)

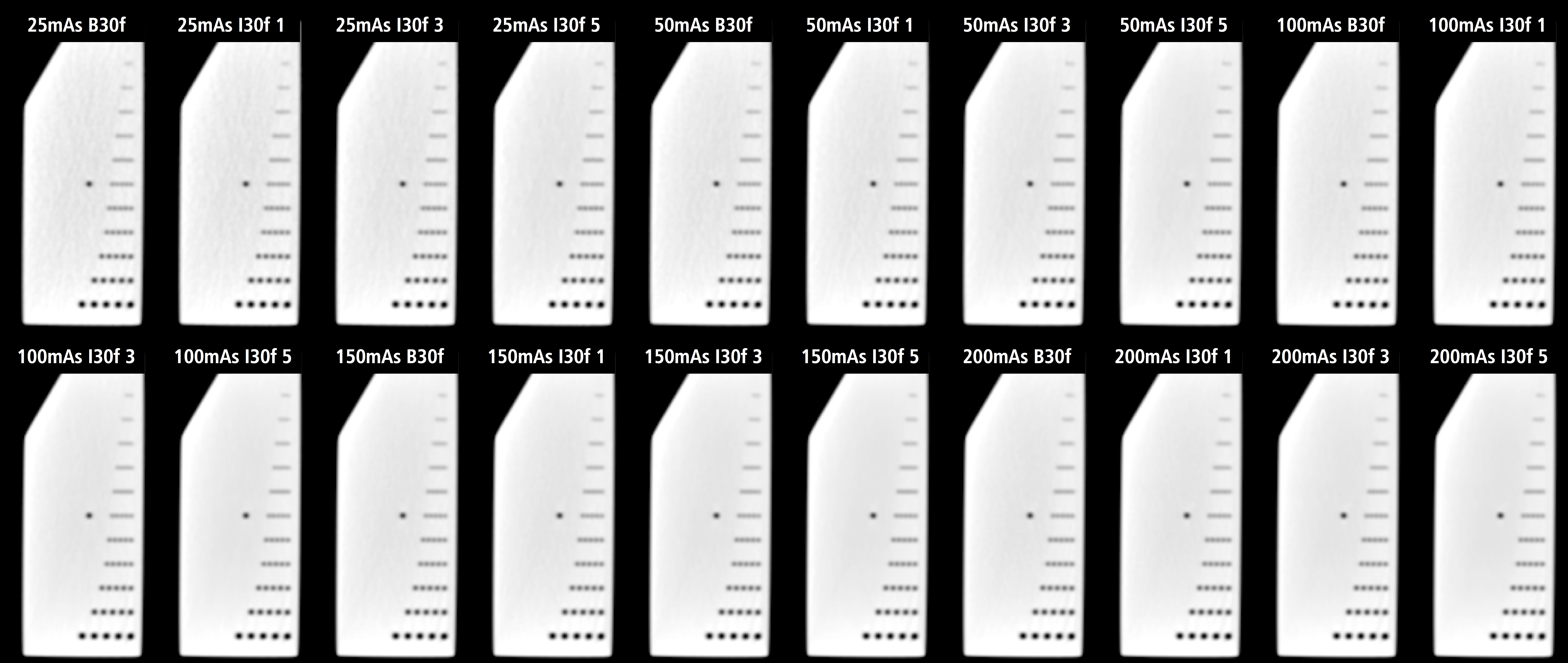

Supplement: Figure S1 — Illustration of spatial resolution in different datasets. Montage of axial slices through the spatial resolution phantom at the same z-position for all 20 datasets acquired at different dose levels and reconstructed using different algorithms and filter strengths. The visual assessment reveals no difference in spatial resolution between the different datasets as confirmed by the quantitative analysis. The reduction of image noise with increasing dose and increase filter strength of the SAFIRE algorithm can be noted in the homogeneous part of the phantom. (TIF) [file pone.0056875.s001.tif]
